# Enhancing Situational Awareness with VAS-Compass Net for the Recognition of Directional Vehicle Alert Sounds

**DOI:** 10.3390/s24216841

**Published:** 2024-10-24

**Authors:** Chiun-Li Chin, Jun-Ren Chen, Wan-Xuan Lin, Hsuan-Chiao Hung, Shang-En Chiang, Chih-Hui Wang, Liang-Ching Lee, Shing-Hong Liu

**Affiliations:** 1 Department of Automatic Control Engineering, Feng Chia University, Taichung 40724, Taiwan; clchin@fcu.edu.tw (C.-L.C.); m1317029@o365.fcu.edu.tw (H.-C.H.); d1055997@o365.fcu.edu.tw (S.-E.C.); d1019590@o365.fcu.edu.tw (C.-H.W.); 2 Department of Medical Informatics, Chung Shan Medical University, Taichung 40201, Taiwan; s1058024@gm.csmu.edu.tw (J.-R.C.); s1058023@gm.csmu.edu.tw (W.-X.L.); s1058016@gm.csmu.edu.tw (L.-C.L.); 3 Department of Computer Science and Information Engineering, Chaoyang University of Technology, Taichung 41349, Taiwan

**Keywords:** hearing impairments, sound alert assistance system, situational awareness, VAS-compass net, edge computing

## Abstract

People with hearing impairments often face increased risks related to traffic accidents due to their reduced ability to perceive surrounding sounds. Given the cost and usage limitations of traditional hearing aids and cochlear implants, this study aims to develop a sound alert assistance system (SAAS) to enhance situational awareness and improve travel safety for people with hearing impairments. We proposed the VAS-Compass Net (Vehicle Alert Sound–Compass Net), which integrates three lightweight convolutional neural networks: EfficientNet-lite0, MobileNetV3-Small, and GhostNet. Through employing a fuzzy ranking ensemble technique, our proposed model can identify different categories of vehicle alert sounds and directions of sound sources on an edge computing device. The experimental dataset consisted of images derived from the sounds of approaching police cars, ambulances, fire trucks, and car horns from various directions. The audio signals were converted into spectrogram images and Mel-frequency cepstral coefficient images, and they were fused into a complete image using image stitching techniques. We successfully deployed our proposed model on a Raspberry Pi 5 microcomputer, paired with a customized smartwatch to realize an SAAS. Our experimental results demonstrated that VAS-Compass Net achieved an accuracy of 84.38% based on server-based computing and an accuracy of 83.01% based on edge computing. Our proposed SAAS has the potential to significantly enhance the situational awareness, alertness, and safety of people with hearing impairments on the road.

## 1. Introduction

When people with hearing impairments walk along a road, they often cannot identify the direction from which vehicles are approaching due to difficulties in hearing or an inability to hear surrounding sounds. This situation makes them exposed to danger when walking along a road. Cheng et al. [1] found that people with hearing impairments were more likely to have traffic accidents, with a 41% higher risk compared with those with normal hearing. Furthermore, a study by Tiwari and Ganveer revealed that approximately 10.2% of traffic accidents resulting in injury or death are due to hearing impairments in the involved individuals [2].

To enhance situational awareness, hearing aids such as amplification aids, cochlear implants, or sound alert aids are commonly used [3]. However, amplification aids face noise issues, while cochlear implants are costly and require surgical implantation. Sound alert aids provide non-auditory alerts to surrounding dangers, thereby enhancing safety and alertness for people with hearing impairments. Recently, the development of deep learning for sound alert aids has been flourishing. Esquivel Ramirez et al. [4] developed a deep learning model using a convolutional neural network (CNN) for alert sound recognition, improving safety and quality of life for hearing-impaired individuals. Veena et al. [5] proposed a deep neural network (DNN) for sound classification to help hearing-impaired individuals to perceive their surroundings better. Yağanoğlu et al. [6] introduced a vibration-based wearable device to enhance environmental perception for hearing-impaired individuals.

In recent years, the application of deep learning technology in the field of sound recognition has made significant progress [7]. Abhay et al. [8] successfully developed a hybrid spectrogram model based on a CNN to classify noise, effectively handling complex sound scenes. Shivam et al. [9] used a CNN combined with Mel-frequency cepstral coefficients (MFCCs) to develop an innovative sound classification and recognition system. Additionally, Chang et al. [10] applied the Transformer model for end-to-end multi-speaker speech recognition in both single- and multi-channel environments. In the study of the direction perception of sound sources, many scholars have successfully used beamforming technology combined with CNNs to estimate the direction of arrival (DOA), paving new paths for the localization technology of sound sources [11,12,13,14]. The advances in this technology directly inspired this research work to explore the further improvement of the accuracy and practicality of sound source identification. Bermant [15] proposed the BioCPPNet architecture based on U-Net, utilizing different biological sound sources for the identification process. Xie et al. [16] utilized the CNN-GAP model with techniques such as harmonic percussive source separation (HPSS), Savitzky–Golay filtering, and nearest neighbor filtering to transform audio signals into multidimensional spectrograms. Tran et al. [17] employed a dual-channel model to capture sound features from various dimensions, allowing for a more comprehensive acquisition of sound information from multiple perspectives. These studies not only demonstrate the efficacy of CNNs in identifying sound categories and directions but also showcase the immense potential of deep learning technologies in handling complex audio data. However, the high demand for computational resources displayed by these technologies may pose limitations in resource-limited environments, especially when real-time processing is required.

To solve the computational constraints of edge devices, recent studies have increasingly applied TinyML technologies and transfer learning to enhance sound recognition and processing capabilities. Tan et al. [18] developed EfficientNet to optimize computational efficiency and power consumption. Howard et al. [19] introduced MobileNet, specifically designed for low-power devices, illustrating the potential for high-performance deep learning algorithms in constrained environments. Han et al. [20] proposed GhostNet, which incorporated ‘ghost features’ technology to reduce computational demands and increase processing speed. These advancements in edge computing have notably expanded the deployment of edge devices in the field of auditory aids, demonstrating effective strategies to minimize model size and computational load without sacrificing performance. Therefore, assistive technology has made great advances in its integration with the Artificial Intelligence of Things (AIoT), and there have been numerous applications of edge devices in hearing aids [21]. Siddique et al. [22] developed a sign-language recognition system using the YOLOv7 Tiny model, which was implemented on Jetson Nano edge devices. Furthermore, Yağanoğlu [23] utilized MFCCs and dynamic time warping (DTW) to enable a Raspberry Pi 4 to have a specialized speech recognition system for hearing-impaired people, solving difficulties of communication. These technological developments not only provide practical benefits for individuals with hearing impairments but also broaden the scope of applications for edge computing.

To enhance model accuracy, numerous studies [24,25,26] have adopted ensemble learning for sound recognition, addressing issues such as missing features, incremental learning, and class imbalance. In this field, some research has introduced fuzzy rank-based ensemble learning to improve performance, including fuzzy logic, which is used to evaluate and rank the performance of each sub-model. This approach has been validated in various applications, such as the detection of cervical cells and the identification of osteoporosis [27,28].

Traditional auditory aids like hearing aids and cochlear implants are beneficial yet flawed, limiting their utility and increasing demand for innovative solutions. Effective sound alert devices are rare, highlighting the challenges for the hearing-impaired in choosing suitable technologies. Moreover, deep learning models identifying sound directions and sources are scarce. While acoustic testing equipment and directional microphones can accurately determine the direction of signals, Pelletier et al. noted that recognized acoustic testing standards generally require specialized hardware and software, which are difficult and expensive for the general public to access. As a result, they proposed a simple and inexpensive microphone recording method [29]. Additionally, the microphone of the Arduino Nano 33 BLE Sense (STMicroelectronics N.V., Switzerland) has been used as a recording device in the fields of sound recognition and classification [30], demonstrating the quality of its sound recording.

In our previous research [31], we presented a wearable device using an EfficientNet-based fuzzy rank-based ensemble model to identify vehicle alert sounds, reducing traffic risks for the hearing-impaired. However, it did not have the ability to recognize and indicate the direction of sounds. This limitation made it difficult for the hearing-impaired to know how to effectively avoid dangers. Therefore, we developed a new edge AI model for a sound alert assistive system (SAAS). This model can alert users to different vehicle alert sounds and indicate their direction of approach—from the left side, from the right side, or from behind.

The main contributions of this study are summarized as follows:

- This study converts self-recorded vehicle alert sounds into an image dataset and makes it publicly available on the Kaggle platform for analysis competitions.
- This study proposes a fuzzy rank-based ensembled model for identifying categories of alert sounds and directions from spectrogram images and MFCC images. This fuzzy-based approach is applied in this field for the first time and achieves significant accuracy.
- Our proposed model in this study is tested on an edge AI device, displaying good performance.
- The proposed SAAS is an innovative application integrating inclusive technology and represents an effective tool for people with hearing impairments to recognize sound sources and directions.

## 2. Materials and Methods

**Figure 1:**
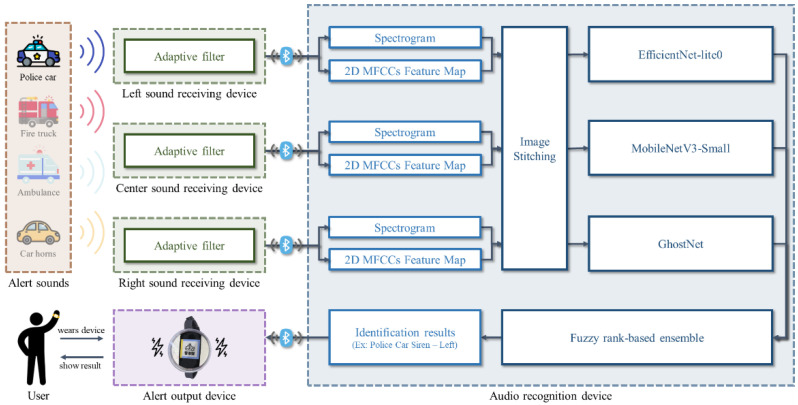
The architecture and the operational process of the proposed SAAS.

As shown in Figure 1, the SAAS developed in this study can be divided into three parts: three devices to record sound signals, an audio recognition device, and an alert output device. The three devices are used for sound reception and preprocessing. The audio recognition device is used to identify the categories of vehicle alert sounds and approaching directions. Finally, the alert output device is used to display the results of the audio recognition device. The following sections will describe the four major parts sequentially: data collection and preprocessing, the construction of VAS-Compass Net, the implementation of the SAAS, and model performance evaluations.

### 2.1. Data Collection and Preprocessing

**Figure 2:**
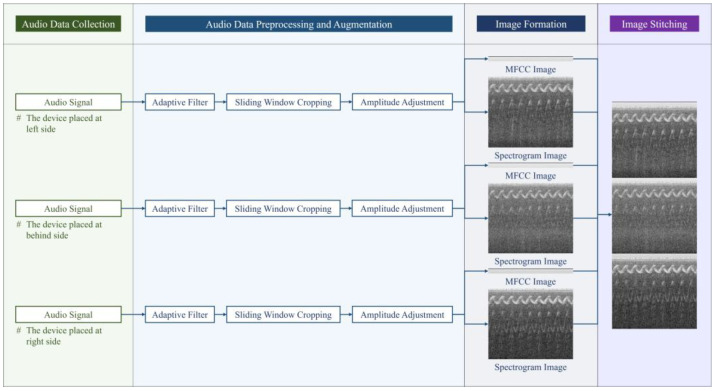
Audio signals are converted to image samples in four steps, including audio data collection, preprocessing and augmentation, image formation, and image stitching. The x-axis of the MFCC images represents the time sequence, and the y-axis represents the 13 MFCCs. The x-axis of the spectrogram images represents the time sequence, and the y-axis represents the spectrum. For a detailed explanation, please refer to Section 3.4.

To obtain the audio samples used for training VAS-Compass Net, this study designed a comprehensive data collection and preprocessing process. As shown in Figure 2, this process consisted of four main steps: audio data collection, audio data preprocessing and augmentation, image formation, and image stitching. The following sections will describe these steps in detail.

#### 2.1.1. Audio Data Collection

This study conducted the collection of audio signals with three devices in Taichung City, Taiwan. The device was designed with an Arduino Nano 33 BLE Sense development board and an MP34DT05-A microphone. The sampling rate was 16 kHz. The user carried a backpack. Three devices were placed on the front, left, and right sides of the backpack. During the recording process, the user walked along a road, and the three devices recorded vehicle alert sounds approaching from approximately 25 m away. The sound sources moved toward the user from the left and right sides and from behind. The user walked along a large road which consisted of a wide open space with no obstructing buildings. Various scenarios were considered, including the user standing still but the sound source moving forward, the user and the sound source both moving forward, and a fixed distance being maintained between the user and the sound source. The sound sources were a police siren, an ambulance siren, a fire truck siren, and a car horn. The background sound in the road was also recorded. Thus, there were 13 categories of audio signals. For one category, the audio signal was measured 20 times, with each measurement lasting 10 s. Therefore, a total of 780 sets (13 types × 20 times × 3 scenarios) were recorded.

#### 2.1.2. Audio Data Preprocessing and Augmentation

After collecting the audio data, the data preprocessing and augmentation steps were carried out to enhance the quality of the data. In the data preprocessing stage, the audio signals were filtered via an adaptive filter to reduce environmental noise and other interferences while retaining the key features of vehicle alert sounds. A sliding window was used to segment the audio signals, with a window size of 3 s and a step size of 1 s, ensuring consistency and continuity in the time series.

**Figure 3:**
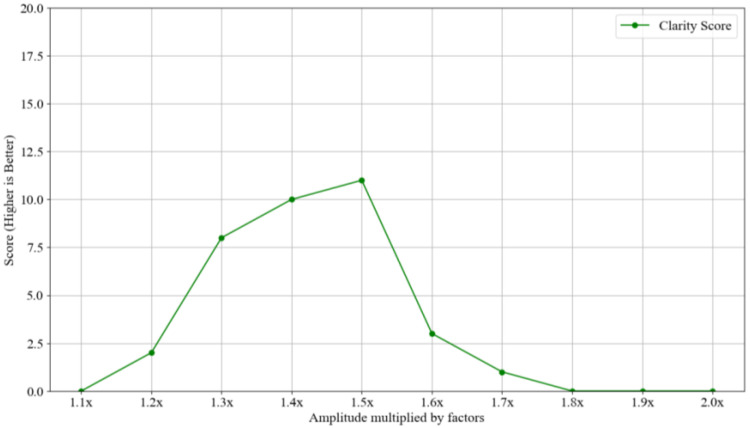
Clarity scores of audio signals at different augmented factors.

Data augmentation was performed to adjust the amplitude of the audio signal. We designed an experiment to evaluate the augmentative factors. We invited 35 participants to listen to these adjusted audio signals and identify them based on both “clarity” and “loudness”. The amplitude of audio signals was multiplied by different factors from 1.1 to 2.0 and increased by 0.1. As shown in Figure 3, when the amplitude was increased 1.5 times, subjects felt the best balance between loudness and clarity. Thus, we chose 1.5 as one factor. Because the score when the factor was 1.4 was only slightly lower than when the factor was 1.5, their augmented data may be close. Thus, we chose 1.3 as another augmented factor. This approach helped to ensure that the augmented dataset was diverse and provided better generalization capabilities for the model. After the preprocessing and augmentation steps, we obtained a total of 6240 samples (260 sets × 8 sliding windows × (2 augmented factors + raw data)).

#### 2.1.3. Image Data Formation

The audio samples were converted into spectrogram images and MFCC images. A short-time Fourier transform (STFT) was used to process the audio signals [32]. The window size for the STFT was 402 points, which corresponded to approximately 25 milliseconds. The step size was 201 points, approximately 12.5 milliseconds. Thus, the spectral number of the STFT was 241 in each audio sample. Each spectrogram image was 241 × 201 pixels in size. The x-axis of these images represented the time sequence, while the y-axis represented the spectrum. The spectrum in each window of STFT was calculated to establish the MFCC, the formulas of which are shown in Equations (1) and (2).

(1) $$S\left[k\right]=log\left(\sum_{n=0}^{N-1}{\left|X\left(n\right)\right|}^{2}H_{k}\left(n\right)\right),$$

where X(n) is the spectral data, $H_{k}\left(n\right)$ is the k-th Mel-scale filter bank, and N is the number of FFT points. $S\left[k\right]$ is the Mel spectral energy, which represents the spectral energy.

(2) $$c\left[m\right]=\sum_{k=0}^{K-1}S\left[k\right]\cos\left(m\left(\frac{\pi }{k}\right)\left(k+\frac{1}{2}\right)\right),$$

where $c\left[m\right]$ is the MFCC after the decomposition of the discrete cosine transform. K is the number of the Mel-scale filter bank, and m typically ranges from 0 to 12, representing different MFCCs. Thus, the size of the MFCC image was 241 × 13 pixels. The x-axis of each image represented the time sequence, while the y-axis represented the spectrum. All images used grayscale. This approach could reduce computational consumption while retaining the necessary information.

#### 2.1.4. Image Stitching

**Figure 4:**
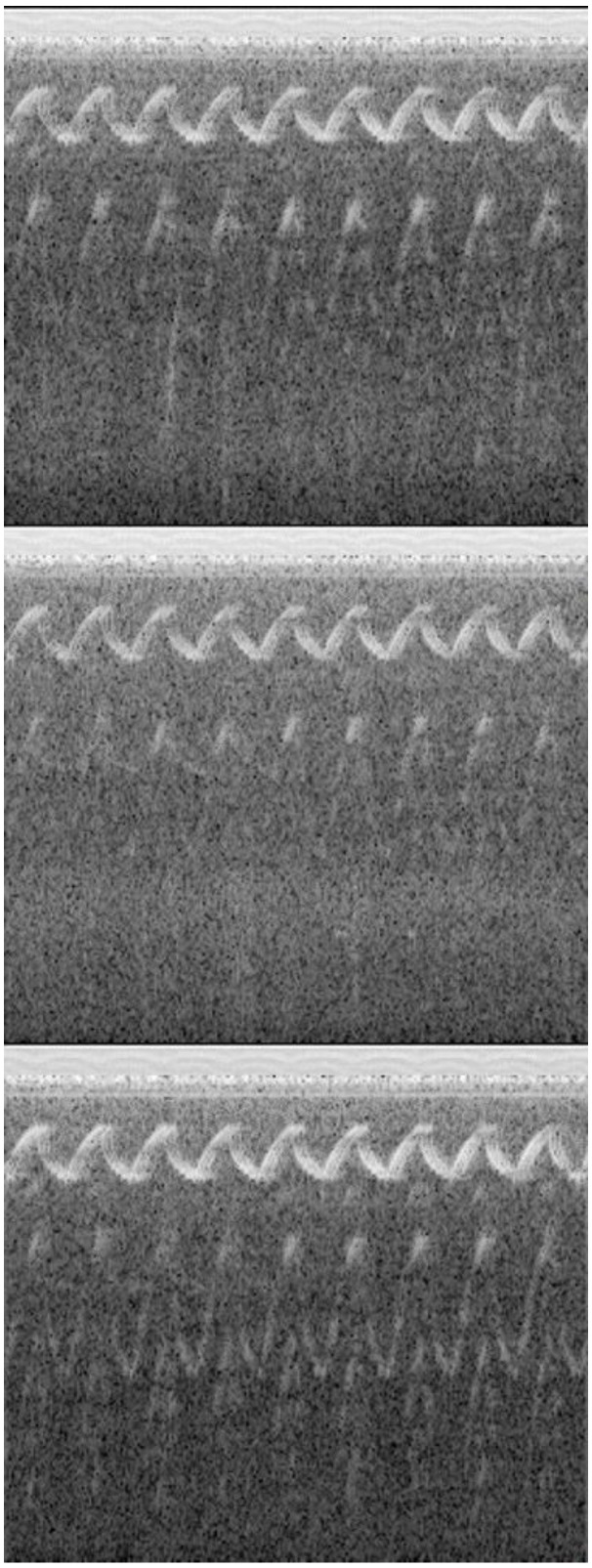
The stitched images include three spectrogram images and three MFCC images, respectively. In each image, the x-axis represents the time sequence, while the y-axis represents the spectrum or spectrum. For a detailed explanation, please refer to Section 3.4.

This study employed image stitching techniques to combine the spectrogram images and MFCC images as the samples. The audio signals were measured using three devices placed on the left and right sides on the back of the user. Thus, there were three spectrogram images and three MFCC images, respectively, in one sample, as shown in Figure 4. This step was crucial for achieving the accurate recognition of sound direction and categories. During the stitching process, the spectrogram images and MFCC images of each audio datum were arranged sequentially: the left MFCC image, the left spectrogram image, the rear MFCC image, the rear spectrogram image, the right MFCC image, and the right spectrogram image. The size of the final stitched image was 241 × 642 pixels. This arrangement not only increased the diversity of input features but also enhanced the model’s ability to recognize sounds from different directions.

After the step of image stitching, this study finally obtained 6240 samples, which were randomly divided into training, validation, and test sets at a ratio of 70%, 10%, and 20% to support the training and performance evaluation of the model. Additionally, these samples were uploaded to the Kaggle platform for public access and download: https://www.kaggle.com/datasets/wanxuanxuanxuan/directional-vehicle-alert-sound-feature-images/data (accessed on 1 April 2024).

### 2.2. VAS-Compass Net

**Figure 5:**
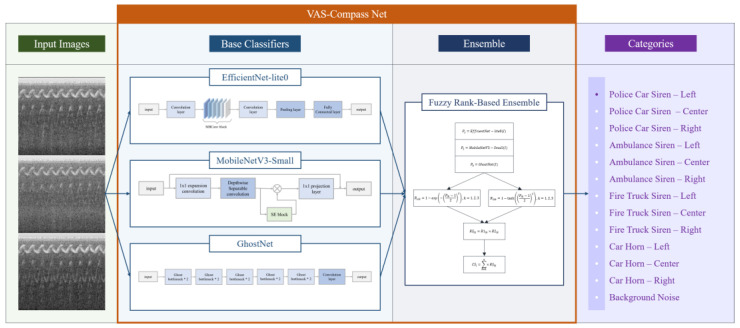
The architecture of VAS-Compass Net.

This study proposed VAS-Compass Net to identify vehicle alert sounds and their directions of approach. Considering the hardware limitations and real-time processing requirements of the audio recognition device in the SAAS, an architecture of a lightweight model was chosen and implemented for edge computing to enhance the efficiency and response speed of audio recognition. The architecture of VAS-Compass Net is shown in Figure 5, which includes the input images, basic classifiers, ensemble method, and categories. The following sections provide a detailed description of the architecture.

#### 2.2.1. Basic Classifiers

The basic classifiers consisted of three lightweight convolutional neural networks: EfficientNet-lite0, MobileNetV3-Small, and GhostNet. Each network independently processed the classification. These models were chosen as the base classifiers because they maintained high performance while having lightweight structures, making them suitable for operation on edge devices with limited computational resources.

- EfficientNet-lite0

EfficientNet-lite0, developed by Tan et al. [18], is an efficient model architecture that simplifies the original EfficientNet design to reduce computational resources. This model removes the Swish activation function and the Squeeze-and-Excitation module, replacing them with the traditional ReLU function, making it more suitable for devices with limited computational resources. The model size is approximately 18 Mbytes, with about 488 million floating-point operations (FLOPs).

- MobileNetV3-Small

The MobileNetV3-Small architecture, developed by Howard et al. [19], optimizes its lightweight structure by combining depth-wise separable convolutions and attention mechanisms, further reducing computational resources while maintaining good performance. This model is specifically tuned to enhance computational speed and efficiency, making it suitable for real-time audio processing applications. The model size is approximately 9.8 Mbytes, with about 60 million FLOPs.

- GhostNet

The GhostNet architecture, developed by Han et al. [20], effectively reduces the number of parameters and computational resources through its innovative Ghost module. This module generates a small number of intrinsic features and then uses simple linear operations to rapidly expand these features, improving overall computational efficiency. The model size is approximately 29.9 Mbytes, with about 141 million FLOPs.

To further reduce the model size and operational efficiency, this study applied pruning and quantization techniques for these three models. Specifically, structured pruning was used to reduce parameters by removing entire convolutional kernels or channels in the neural network layers, effectively reducing the model size while maintaining performance [33,34].

#### 2.2.2. Ensemble Method

Ensemble learning is a machine learning strategy that combines the predictions of multiple models to improve accuracy and stability. In this study, we used three base classifiers: EfficientNet-lite0, MobileNetV3-Small, and GhostNet. However, due to the high complexity and variability of the task of recognizing vehicle alert sounds, the performance of these models when processed independently is limited. Therefore, we chose to integrate the outputs of these three models through a fuzzy rank-based ensemble method to achieve higher accuracy and better stability.

In the VAS-Compass Net architecture, a fuzzy rank-based model is used to integrate the outputs from EfficientNet-lite0, MobileNetV3-Small, and GhostNet and to calculate fuzzy rankings for each category. This model utilizes exponential and hyperbolic tangent functions to transform the estimated weights for P_ik_ of each category, generating two distinct fuzzy ranking scores, R_1_ and R_2_, as shown in Equations (3) and (4). Then, the two fuzzy ranking scores are multiplied to produce a combined ranking score, RS_ik_, for each category i, as depicted in Equation (5). Finally, to compute the composite confidence score, CS_i_, for each category, the model sums the combined ranking scores across all three models, as indicated in Equation (6).

(3) $$R_{1ik}=1-exp(-{\left(\frac{P_{ik}-1}{2}\right)}^{2}),\;k=1,2,3.$$

This reflects the difference between the estimated weight for category i in model k and the ideal weight (where 1 is optimal).

(4) $$R_{2ik}=1-tanh(-{\left(\frac{P_{ik}-1}{2}\right)}^{2}),k=1,2,3.$$

This considers a nonlinear adjustment for weight bias to smooth the impact of outliers.

(5) $${RS}_{ik}={R1}_{ik}\times {R2}_{ik}.$$

This score integrates two different measures of uncertainty to achieve a more refined evaluation of the category.

(6) $${CS}_{i}=\sum_{k=1}^{3}\times \;{RS}_{ik}$$

This confidence score provides a quantified indicator of the overall confidence level in the classification. These scores were then used to produce the final confidence score for each category. The winner was the category of classification.

### 2.3. Implementation of the SAAS

This section describes the composition and operation process of the SAAS, which consists of three sound-receiving devices, an audio recognition device, and an alert output device. The sound-receiving devices, comprising an Arduino Nano 33 BLE Sense development board and an MP34DT05-A microphone, received and processed the sounds around the user. Within this microcontroller, we designed an adaptive filter to effectively enhance critical alert signals while suppressing irrelevant background noise. The preprocessed audio signal was converted into spectrogram images and MFCC images, which were transmitted via a Bluetooth module to the audio recognition device for proceeding with further classification.

The core of the audio recognition device was a Raspberry Pi 5 microcomputer (Raspberry Pi Foundation, United Kingdom). It first used the technique of image stitching mentioned in Section 2.1.4 to combine the spectrogram images and MFCC images into one image. This image was then inputted into VAS-Compass Net for audio recognition. Once the alert sound and direction were identified, the relevant information was immediately transmitted via the Bluetooth module to the alert output device to activate the corresponding alert mechanism.

**Figure 6:**
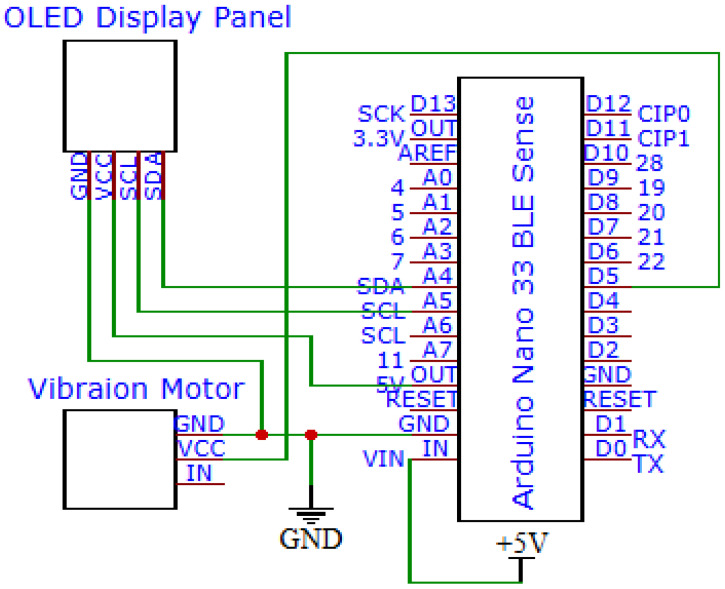
The circuit diagram of the alert output device in the SAAS.

The alert output device was a wearable aid composed of an Arduino 33 BLE Sense microcontroller, a vibration module, and an OLED display panel, designed in the form of a watch. Figure 6 shows the connections between the Arduino 33 BLE Sense microcontroller, the vibration module, and the OLED display panel. When the target audio is successfully identified by the audio recognition device, this device alerts the user by activating the vibration module and displaying the category of the alert sound and its direction of approach on the OLED panel, providing immediate and intuitive guidance.

**Figure 7:**
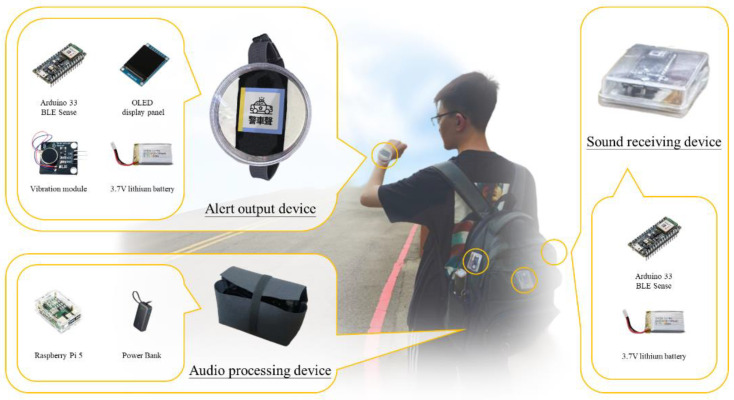
The main hardware components of the SAAS and their ideal placement on the user’s body.

Figure 7 illustrates the hardware components of the sound-receiving devices, the audio recognition device, and the alert output device, along with their ideal positions on the user. This configuration ensures the maximization of sound reception and the effective transmission of alert messages. The size of the sound-receiving devices is 5.5 cm × 4.5 cm × 1.5 cm, making them suitable for putting on a backpack or a user’s arms and back. The size of the audio recognition device is 11.0 cm × 6.5 cm × 7.5 cm, suitable for placement in a backpack or pocket. Finally, the size of the alert output device is 6.0 cm × 6.0 cm × 3.25 cm and can be worn on the wrist.

### 2.4. Model Performance Evaluations

As shown in Table 1, this study used a confusion matrix to illustrate the performance of the VAS-Compass Net model in the classification of vehicle alert sounds and their source directions. The confusion matrix comprised four parts: true positive (TP), false positive (FP), true negative (TN), and false negative (FN). TP indicates the number of positive instances correctly identified by the model, TN indicates the number of negative instances correctly excluded by the model, FP indicates the number of negative instances incorrectly predicted as positive by the model, and FN indicates the number of positive instances incorrectly predicted as negative by the model. Additionally, to comprehensively evaluate the performance of VAS-Compass Net, this study selected accuracy, recall, precision, and F1 score as the main evaluation metrics.

## 3. Results

This section presents the experimental setup, model training and evaluation process, and a detailed analysis of VAS-Compass Net’s performance in different computing environments. We compare the experimental results of the model’s application on both server-end and edge devices. This study aims to explore the performance of the model in handling complex sound recognition and the contribution of the fuzzy rank-based ensemble model to improving the accuracy of recognition. Moreover, we examine the power consumption during actual execution in the device to evaluate its feasibility in practical applications.

### 3.1. Experimental Setup and the Hyperparameters for Model Training

The system configuration in the study used an Intel^®^ Xeon^®^ W5-2455X processor, which is manufactured by Intel Corporation in Santa Clara, CA, USA, an NVIDIA RTX A6000 GPU, 128 GB of RAM, and 9 TB of storage space. During model training, we set the batch size to 64 and the training epochs to a maximum of 100 for each model. Additionally, we introduced an early stopping mechanism to halt training when the performance on the validation set showed improvement for several consecutive epochs. The learning rate was 0.001, categorical cross-entropy loss was selected as the loss function, and the Adam optimizer was used for parameter updates. VAS-Compass Net was built using Python 3.8.12 and developed and trained with the PyTorch deep learning framework, providing high flexibility and scalability for model implementation.

### 3.2. Performance of VAS-Compass Net in Server-Based Environments

**Figure 8:**
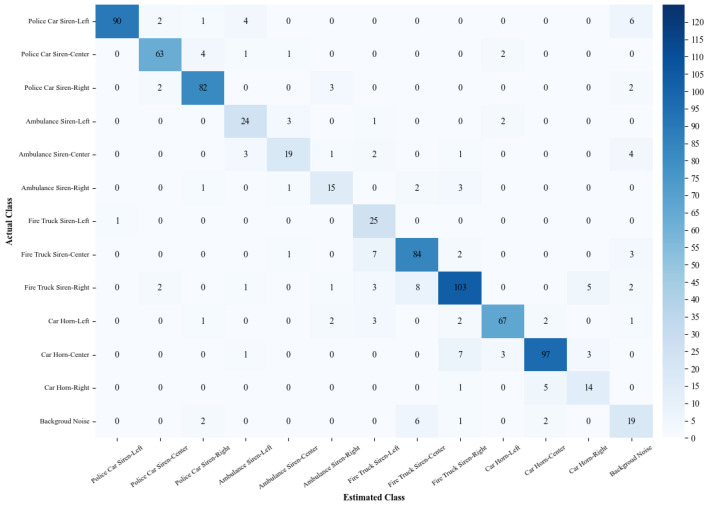
The confusion matrix of VAS-Compass Net in the server-based environment.

VAS-Compass Net, deployed in the SAAS of this study, is an integrated model combining three 2D CNNs with a fuzzy ranking ensemble technique. In a server-based computing environment, the model was trained, validated, and tested on a medium-sized dataset containing 6240 samples, with 1248 data points randomly selected as the test set to evaluate the model’s performance. The performance is illustrated in the confusion matrix in Figure 8. The model achieved an accuracy of 84.38%, a recall of 84.38%, a precision of 85.44%, and an F1 score of 84.66%.

### 3.3. Performance of VAS-Compass Net in SAAS Deployments

**Figure 9:**
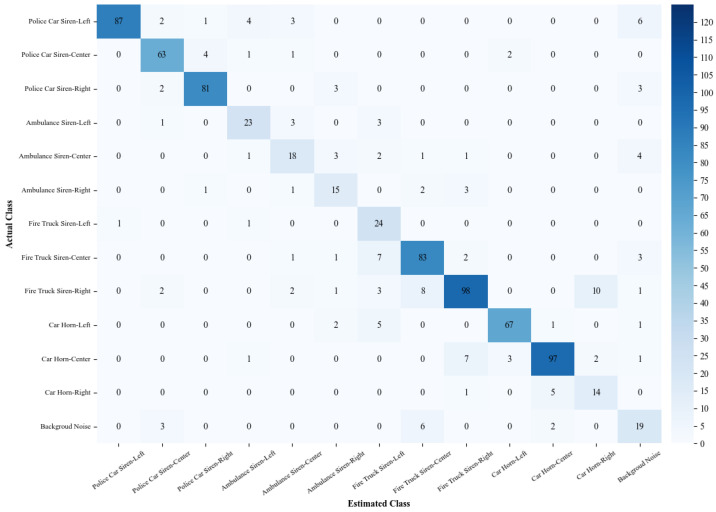
The confusion matrix of VAS-Compass Net in the edge computing device.

The proposed SAAS is an edge computing device using a Raspberry Pi 5 microcomputer as the core computing unit. We deployed the VAS-Compass Net model in this system and enabled it to receive preprocessed audio signals to identify sound categories and directions. The confusion matrix in Figure 9 illustrates the performance of VAS-Compass Net in recognizing the categories and directions of vehicle alert sounds in the edge computing environment. In terms of performance metrics, the model achieved an accuracy of 83.01%, a recall of 83.01%, a precision of 84.72%, and an F1 score of 83.52%

Based on the experimental results, this study found that the performance of VAS-Compass Net in edge computing is comparable to its performance based on server-based computing. As shown in the performance comparison analysis in Table 2, VAS-Compass Net performed well across all metrics. The accuracy, recall, precision, and F1 score of VAS-Compass Net based on edge computing were only 1.37%, 1.37%, 0.72%, and 1.14% lower than those based on server-based computing, respectively.

### 3.4. The Results of the Feature Concatenation

**Figure 10:**
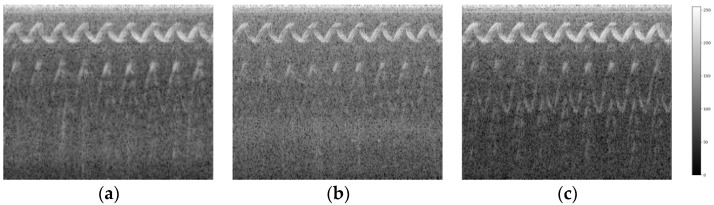
Spectrogram images of a police car siren collected by the device (**a**) on the left side, (**b**) at the rear, and (**c**) on the right side. In each spectrogram image, the x-axis represents the time sequence and the y-axis represents the spectrum.

In this study, audio signals were collected from complex environments using sound-receiving devices placed on the left, right, and front sides of a backpack. After noise filtering, the signals were converted into spectrograms. In addition, sound features are shown in the spectrogram images and MFCC images. The spectrogram images of the sound of a police siren in Figure 10a–c are the audio signals recorded by the devices placed on the left, rear, and right sides, respectively. The sound source was on the left side of the user. The x-axis of the image represents the time sequence, and the y-axis represents the spectrum. The unique and regular wave patterns in these spectrogram images represent the characteristics of the sound of a police siren. The wave patterns in Figure 10a are clearer than those in the other images, indicating that the sound source is closer to the sound-receiving device. The relatively lighter gray and less prominent frequency components in Figure 10b indicate a reduction in the energy of the siren sound due to the orientation of the sound waves and an increase in the energy of background noise. In Figure 10c, the other waves at a lower frequency indicate interference from other sound sources or noise. This shows that the recording device is farther from the sound source, leading to an increased proportion of environmental noise.

**Figure 11:**
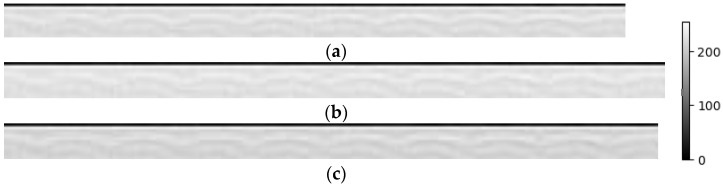
MFCC images of a police car siren collected by the device (**a**) on the left side, (**b**) at the rear, and (**c**) on the right side. In each MFCC image, the x-axis represents the time sequence and the y-axis represents the spectrum.

Figure 11a–c show the MFCC images. The device and sound source are the same as in Figure 10. The x-axis represents the time sequence, and the y-axis represents the 13 MFCCs. The gray intensity in the images indicates the energy levels at specific times and spectra, with white representing high energy and black representing low energy. In three MFCC images, a distinct black band can be observed at the top of the images, indicating relatively weak energy at these times and spectra. Other parts are shown in shades of gray to white, indicating increasing energy levels. This gray variation helps us to understand the dynamic changes in time and spectrum within the audio. The gray changes indicate a segment of consistent frequency in the sound of the police siren. Additionally, from the overall lighter color and lower contrast in Figure 11a compared to Figure 11b,c, it is evident that the rear device captured more sound.

### 3.5. The Impact of Image Stitching Arrangement

This section explores and compares the impact of different arrangements of spectrogram images and MFCC images on the accuracy of VAS-Compass Net. In the experiment, four different arrangement methods were designed to stitch the images, and the stitched images were input into the model for training. Four image stitches based on differently arranged sequences of spectrogram images and MFCC images in the horizontal and vertical directions were used.

**Figure 12:**
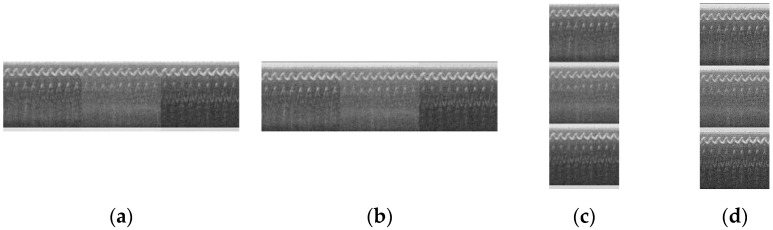
Different stitching images for spectrogram images and MFCC images: (**a**) Spectral_H, (**b**) MFCC_H, (**c**) Spectral_V, and (**d**) MFCC_V.

- Spectral_H: The spectrogram image is placed above the MFCC image, and the arranged sequence in the horizontal direction comprises images from the left side, from behind, and from the right side, respectively, as depicted in Figure 12a. The size of the image stitching is 723 × 214.
- MFCC_H: The MFCC image is placed above the spectrogram image, and the arranged sequence in the horizontal direction comprises images from the left side, from behind, and from the right side, as depicted in Figure 12b. The size of the image stitching is 723 × 214.
- Spectral_V: The spectrogram image is placed above the MFCC image, and the arranged sequence in the vertical direction comprises images from the left side, from behind, and from the right side, as depicted in Figure 12c. The size of the image stitching is 241 × 642.
- MFCC_V: The MFCC image is placed above the spectrogram image, and the arranged sequence in the vertical direction comprises images from the left side, from behind, and from the right side, as depicted in Figure 12d. The size of the image stitching is 241 × 642.

Table 3 shows the test results of the model with different stitching images. The model has the best performance with MFCC_V images, with an accuracy of 84.38%, a recall of 84.38%, a precision of 85.44%, and an F1 score of 84.66%.

### 3.6. Recognition Results Displayed by the Wearable Assistive Devices

**Figure 13:**
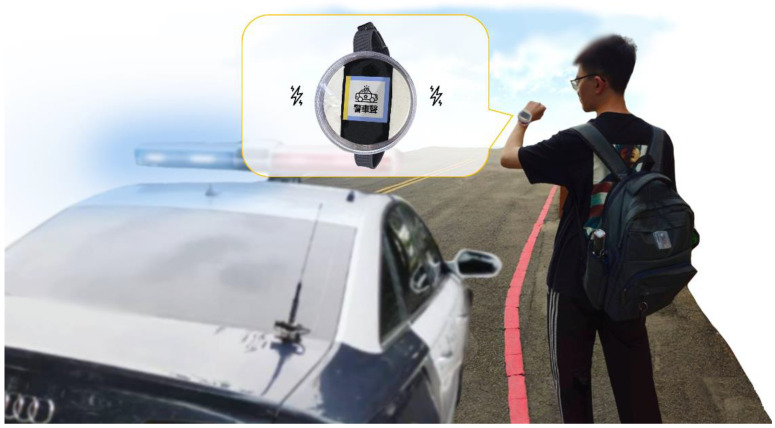
The SAAS indicates a police siren approaching the user from the left side.

Deploying the trained VAS-Compass Net into the SAAS enabled the identification of vehicle alert sounds and directions. When the model successfully identified the target sound and direction, the system immediately alerted the user through the vibration module on the SAAS. Simultaneously, the category of alert sound was displayed on the OLED panel, and the direction of sound source was indicated by a bright yellow marker on the screen edge. Figure 13 shows an example where a police siren is identified, and the direction of the sound source is indicated by a bright yellow marker on the screen edge. This indicates that a police vehicle is approaching from the user’s left side.

### 3.7. Satisfaction Survey

To comprehensively evaluate the actual impact of the SAAS on different user groups, this study employed a random sampling method, inviting 273 participants to experience the system and complete a questionnaire on aspects such as convenience, necessity, appeal, and practicality. The respondents included 135 individuals with hearing impairments and 138 individuals without hearing impairments, aged 18 to 65, with 52% being female and 48% being male. The questionnaire content thoroughly assessed the participants’ usage experiences and evaluations of the system’s functions.

To analyze the differences in opinions among different groups, an independent sample t-test was conducted. The results show a significant difference in the perception of the system’s effectiveness in identifying alerts while driving or walking along a road between hearing-impaired and non-hearing-impaired individuals (t = 2.35, *p* < 0.05), with hearing-impaired individuals showing significantly higher agreement. Moreover, approximately 82% of respondents supported the view that the SAAS could enhance safety. Respondents also provided numerous suggestions and expectations regarding the system’s functional design and wearing comfort, which will be valuable for future product improvement and optimization.

### 3.8. Power Consumption Analysis

In this study, we meticulously recorded the power consumption of VAS-Compass Net during both training and operation on the server-end device, as well as during operation on the edge device, the Raspberry Pi 5 microcomputer. For the server-side device, we used a power meter to monitor the real-time power consumption during the model’s training and inference phases. Moreover, we employed a USB power monitor to precisely measure the power consumption of the Raspberry Pi 5 during its operation. During training, the server consumed an average of 850 watts/h, while during inference, the consumption dropped to 600 watts/h. In contrast, the Raspberry Pi 5 consumed an average of 15 watts/h over a continuous four-hour operation period. In total, it consumed 60 watts/h, equivalent to 12,000 milliamps/h (mAh).

## 4. Discussion

This study explored the performance of VAS-Compass Net and compared the proposed model with other existing technologies to highlight its differences and advantages. Finally, we mention the limitations of this study.

### 4.1. Comparison with Other Models

To evaluate the performance of VAS-Compass Net, we compared it with other methods in the tasks of alert sound recognition and direction identification. Manna et al. [27] developed an ensemble model combining three large CNN architectures, which, despite capturing fine features, demand excessively high computational resources, making it unsuitable for deployment on edge computing devices. In contrast, the models proposed by Tan [18], Howard [19], and Han [20]—EfficientNet-lite0, MobileNetV3-Small, and GhostNet, respectively—are designed specifically for edge computing. However, to balance operational efficiency and performance, these models typically sacrificed some recognition accuracy.

The proposed VAS-Compass Net combines the advantages of the aforementioned lightweight models with the fuzzy rank-based ensemble method and was successfully applied in our designed SAAS. As shown in Table 4, VAS-Compass Net significantly outperforms the other lightweight models, such as EfficientNet-lite0, MobileNetV3-Small, and GhostNet in terms of recognition effectiveness. Its accuracy and F1 score reach 89.61% and 87.35%, respectively, surpassing the three lightweight models. Additionally, VAS-Compass Net achieves an average inference time of only 8.75 milliseconds, which is significantly lower than the ensemble model proposed by Manna et al. [27]. These results not only demonstrate our model’s ability to have fast processing speed and low resource consumption but also highlight its excellent recognition performance, indicating its vast potential for SAAS applications.

### 4.2. Performance Analysis Between Edge Computing and Server-Based Computing

A comparison of the performance of VAS-Compass Net based on edge computing and server-based computing is shown in Table 3. Although VAS-Compass Net demonstrates excellent performance in the SAAS, there is a slight decline compared to server-side computing. This phenomenon is mainly due to the following two factors:

- Hardware capability differences: While the computational power of edge computing devices has significantly improved in recent years, their processing capabilities, memory capacity, and data processing speed are still limited compared to dedicated servers. These limitations can affect the model’s ability to process complex data, especially in real-time data stream processing scenarios.
- Environmental and operational conditions: Edge computing devices are often deployed in variable environments, which can affect their operational stability and computational efficiency due to external factors such as temperature, humidity, and mechanical vibrations.

Despite these challenges, the implementation of VAS-Compass Net in the edge computing device displayed significant potential, particularly in applications requiring a rapid response and constrained resource environments. This study demonstrated that even with limitations in hardware capabilities and operational environments, our model could effectively run and achieve performance close to that of server-based computing. This experiment not only showcased the adaptability of VAS-Compass Net but also provided valuable experience for future deployments of edge computing technology in more challenging environments. Future work will focus on further optimizing the model’s power computation to enable broader commercial and safety applications.

### 4.3. Energy Consumption Analysis and Optimization

While VAS-Compass Net demonstrated high accuracy in vehicle alert sound recognition tasks, its power efficiency in edge computing environments requires further optimization to support extended daily use. During more than 4 h of continuous operation, the Raspberry Pi 5 consumed an average of 15 watts/h, totaling 60 watt/h of energy, equivalent to approximately 12,000 mAh of battery capacity. This power consumption indicates that if the SAAS were to run continuously for 24 h, it would require about 72,000 mAh of battery capacity.

To satisfy these power demands, future work will explore several directions:

- Dynamic power management (DPM): Implementing DPM includes intelligently switching the processor’s power states to adapt to different computational load demands, thereby reducing power consumption during low-load periods.
- Development of more efficient data processing algorithms: Specifically, optimizing algorithms for sound processing and feature extraction to reduce the necessary computational steps and increase processing speed.
- Utilizing more energy-efficient hardware components: Low-power processors and optimized memory management are considered to enhance overall energy efficiency.

These adjustments aim to extend the device’s operational time without compromising performance, thereby enhancing the system’s practicality for continuous daily use in real-world environments.

### 4.4. Limitations of This Study

According to the data collection, this study has three limitations. First, in the experiment, the user carried a backpack. Three devices were place on the left, front, and right sides of backpack to record the audio signals. The sound sources came from the left and right sides and from behind. Since most people will notice the situation in front of them while moving, we did not record sound sources coming from the front side. However, nearby buildings can cause our system to experience misjudgment due to sound refraction. Second, the audio sounds were recorded in Taichung City, Taiwan. Thus, the proposed SAAS can be suitably used in Taiwan. Although the SAAS may not be applicable in other countries, our model can still serve as a pre-trained model to accelerate development elsewhere. Third, the acoustic environment was a wide open space with no obstructing buildings. We did not consider the scenario of sound reflecting from buildings.

## 5. Conclusions

In this study, we proposed an SAAS that uses VAS-Compass Net to recognize the directions and categories of vehicle alert sounds, assisting individuals with hearing impairments. VAS-Compass Net was deployed on a microcomputer for edge computing, with the recognition results displayed on an OLED panel and using a vibration module to alert the user. VAS-Compass Net achieved an accuracy of 84.38% based on server-based computing and 83.01% based on edge computing, demonstrating its reliability and practicality in real-time applications. The SAAS with VAS-Compass Net showed exceptional performance in the classification of the categories and directions of vehicle alert sounds, and it displayed significant potential for future development. Future research will focus on expanding this technology’s application and improving its accuracy and reliability under various environmental conditions.

## Figures and Tables

**Table 1:** Confusion matrix.

	Estimated Class		
Actual class		**Positive**	**Negative**
	Positive	TP (True Positive)	FN (False Negative)
	Negative	FP (False Positive)	TN (True Negative)

**Table 2:** Comparative performances of VAS-Compass Net based on server-based computing and edge computing.

VAS-Compass Net	Accuracy (%)	Recall (%)	Precision (%)	F1 Score (%)
Server-based computing	84.38	84.38	85.44	84.66
Edge computing	83.01	83.01	84.72	83.52

**Table 3:** Comparative performances of different stitching images.

Arrangement of Images	Accuracy (%)	Recall (%)	Precision (%)	F1 Score (%)
Spectral_H	78.13	74.07	82.05	78.12
MFCC_H	78.73	76.39	81.48	78.62
Spectral_V	82.93	78.70	87.18	82.68
MFCC_V	84.38	84.38	85.44	84.66

**Table 4:** Comparative results of different classification models.

Method	Accuracy (%)	F1-Score (%)	Average Inference Time (ms)
EfficientNet-lite0 [18]	72.27%	71.52%	5.63
MobileNetV3-Small [19]	68.83%	70.28%	0.72
GhostNet [20]	67.23%	65.93%	1.78
A fuzzy rank-based ensemble of CNN models including Inception v3, Xception, and DenseNet-169 [27]	84.38%	84.66%	10.12
VAS-Compass Net	89.61%	87.35%	8.75

## Data Availability

https://www.kaggle.com/datasets/wanxuanxuanxuan/directional-vehicle-alert-sound-feature-images/data (accessed on 1 April 2024).
